# Application of a 3D Fusion Model to Evaluate the Efficacy of Clear Aligner Therapy in Malocclusion Patients: Prospective Observational Study

**DOI:** 10.2196/67378

**Published:** 2025-01-15

**Authors:** Chaofeng Liu, Yan Liu, Chunyan Yi, Tao Xie, Jingjun Tian, Peishen Deng, Changyu Liu, Yan Shan, Hangyu Dong, Yanhua Xu

**Affiliations:** 1 Yunnan Key Laboratory of Stomatology Department of Orthodontics Kunming Medical University & Affiliated Stomatological Hospital Kunming China; 2 Yunnan Key Laboratory of Stomatology Department of Second Clinic Kunming Medical University & Affiliated Stomatological Hospital Kunming China; 3 Department of Stomatology Kunming Medical University & Affiliated Yan'an Hospital Kunming China

**Keywords:** clear aligners, CBCT, intraoral scanning, fusion model, artificial intelligence, efficacy evaluation, orthodontic treatment

## Abstract

**Background:**

Investigating the safe range of orthodontic tooth movement is essential for maintaining oral and maxillofacial stability posttreatment. Although clear aligners rely on pretreatment digital models, their effect on periodontal hard tissues remains uncertain. By integrating cone beam computed tomography–derived cervical and root data with crown data from digital intraoral scans, a 3D fusion model may enhance precision and safety.

**Objective:**

This study aims to construct a 3D fusion model based on artificial intelligence software that matches cone beam computed tomography and intraoral scanning data using the Andrews’ Six Element standard. The model will be used to assess the 3D effects of clear aligners on tooth movement, to provide a reference for the design of pretreatment target positions.

**Methods:**

Between May 2022 and May 2024, a total of 320 patients who completed clear aligner therapy at our institution were screened; 136 patients (aged 13-35 years, fully erupted permanent dentition and periodontal pocket depth <3 mm) met the criteria. Baseline (“simulation”) and posttreatment (“fusion”) models were compared. Outcomes included upper core discrepancy (UCD), upper incisors anteroposterior discrepancy (UAP), lower Spee curve deep discrepancy (LSD), upper anterior teeth width discrepancy (UAW), upper canine width discrepancy (UCW), upper molar width discrepancy (UMW), and total scores. Subanalyses examined sex, age stage (adolescent vs adult), and treatment method (extraction vs nonextraction).

**Results:**

The study was funded in May 2022, with data collection beginning the same month and continuing until May 2024. Of 320 initial participants, 136 met the inclusion criteria. Data analysis is ongoing, and final results are expected by late 2024. Among the 136 participants, 90 (66%) were female, 46 (34%) were male, 64 (47%) were adolescents, 72 (53%) were adults, 38 (28%) underwent extraction, and 98 (72%) did not. Total scores did not differ significantly by sex (mean difference 0.01, 95% CI –0.13 to 0.15; *P*=.85), age stage (mean difference 0.03, 95% CI –0.10 to 0.17; *P*=.60), or treatment method (mean difference 0.07, 95% CI –0.22 to 0.07; *P*=.32). No significant differences were found in UCD (mean difference 0.001, 95% CI –0.02 to 0.01; *P*=.90) or UAP (mean difference 0.01, 95% CI –0.03 to 0.00; *P*=.06) by treatment method. However, adolescents exhibited smaller differences in UCD, UAW, UCW, and UMW yet larger differences in UAP and LSD (df=134; *P*<.001). Extraction cases showed smaller LSD, UAW, and UCW but larger UMW differences compared with nonextraction (df=134; *P*<.001).

**Conclusions:**

The 3D fusion model provides a reliable clinical reference for target position design and treatment outcome evaluation in clear aligner systems. The construction and application of a 3D fusion model in clear aligner orthodontics represent a significant leap forward, offering substantial clinical benefits while establishing a new standard for precision, personalization, and evidence-based treatment planning in the field.

**Trial Registration:**

Chinese Clinical Trial Registry ChiCTR2400094304, https://www.chictr.org.cn/hvshowproject.html?id=266090&v=1.0

## Introduction

### Background

Alveolar bone remodeling is closely linked to orthodontic treatment. Investigating the safe range of orthodontic tooth movement is crucial for the stability of the oral and maxillofacial system following such treatment. Previous studies aimed at evaluating tooth alignment have primarily focused on clinical crown alignment, often neglecting root alignment, except when using panoramic x-rays to assess root parallelism. However, panoramic x-rays exhibit limited accuracy in evaluating the relationship between roots and the surrounding alveolar bone [1,2].

#### Cone Beam Computed Tomography and 3D Digital Models

Cone beam computed tomography (CBCT) has emerged as the most widely used imaging technique capable of effectively distinguishing between soft and hard tissues, and it has been extensively applied in orthodontic treatment. Tsukiboshi et al [3] used CBCT images from 28 patients, generating skull and mandible images through surface rendering, and established standard ranges for these surfaces, thereby allowing clinicians to reliably quantify and visualize patients’ 3D hard tissues of the face. While complete tooth models can be reconstructed from CBCT images, the accuracy of this data ranges from 0.1 to 0.5 mm, and the precision, particularly for the crown portion, does not meet clinical requirements [4-6]. In recent years, digitally reconstructed 3D dental models have demonstrated high accuracy when compared with traditional plaster models, along with the added benefit of reproducible measurements [7-9]. However, these models cannot capture data on the cervical and root portions of the teeth. Therefore, we speculate that fusing the CBCT-obtained data of the necks and roots of the teeth with intraoral scanning (IOS) data of the crowns may address this limitation.

#### Clear Aligner and Andrews Six Elements

Clear aligner orthodontic technology uses a goal-oriented pretreatment target position model; however, the effects of this technology on periodontal hard tissues during orthodontic treatment remain uncertain. Existing research has proposed several methods for evaluating orthodontic treatment outcomes, including the American Board of Orthodontics Objective Grading System and the Peer Assessment Rating Index [10,11]. Based on Angle’s standard of ideal normal occlusion, Andrews conducted a study in the 1960s that examined the natural dentition of 120 permanent teeth that had not undergone orthodontic treatment. The six standards of normal occlusion represent the optimal natural state of occlusion and serve as the objectives of orthodontic treatment. Meanwhile, Andrews defined 6 standards of normal occlusion based on Angle’s principles, encompassing 6 aspects (Textbox 1), which set treatment targets for teeth, dental arches, jaw relationships, and facial esthetics [12]. Previous studies and some scholars have indicated that Andrews’ Six Elements possess advantages in assessing the effectiveness of orthodontic treatment, while the 3D positioning of treatment target positions has also proven effective [13,14].

**Elements.**

• Element I: ideal dental arch (shape, length, crown torque, root position, Spee curve depth, surface contact)

• Element II: ideal front-to-back jaw relationship

• Element III: ideal horizontal jaw positioning

• Element IV: ideal vertical jaw positioning

• Element V: ideal chin protrusion

• Element VI: ideal occlusal relationship

### Objectives

This study aims to develop a 3D fusion model that integrates CBCT data of the cervical and root portions with IOS data of the crown portions, using AI software per Andrews’ Six Elements. We hypothesize that this fusion model will accurately assess the 3D effects of clear aligners on tooth movement, providing a reliable reference for designing pretreatment target positions and ultimately enhancing the precision and safety of orthodontic treatments.

## Methods

### Participant Recruitment

This study was funded in May 2022. We are randomly recruiting patients who have completed orthodontic treatment using clear aligners at the Department of Orthodontics of Kunming Medical University Affiliated Stomatological Hospital. As of the submission of this paper, 320 patients have been recruited. To ensure diversity, patients were purposefully stratified according to sex, age, ethnicity, treatment methods, and types of appliances. Members of the research team invited patients to participate in person. All patients had signed informed consent forms for orthodontic treatment, allowing the use of their anonymized data for research purposes. The collected data included digital intraoral scan models, x-ray images, and clinical photographs. The digital 3D dental models were exported from the iTero Element system and saved in standard template library (STL [3D SYSTEMS]) format. All patient data were encrypted and securely stored in the cloud to ensure confidentiality. Data collection and processing took place from May 2022 to May 2024.

### Sample Selection

Inclusion criteria encompassed individuals aged 13 to 35 years with fully erupted teeth, a good periodontal condition, and a periodontal pocket depth lower than 3 mm. Exclusion criteria included individuals with lip and palate clefts, craniofacial deformities, or skeletal discrepancies that required orthognathic surgery. Based on these criteria, a total of 136 individuals were selected.

### Model Construction

#### Materials and Software

This study used a CBCT scanner (NewTom VG, Aperio Services) to obtain detailed images of the maxillofacial region, along with IOS (iTero Element 2, Align Technology) to capture high-resolution 3D models of the dentition. The software used included the Mimics Innovation Suite (Version 23.0; Materialise) for processing the CBCT data, Geomagic Wrap (Version 2023.0; 3D Systems) for data fusion and model refinement, and specialized orthodontic planning software for computer-based tooth arrangement according to Andrews’ Six Elements diagnostic system.

#### Protocol Steps

**Figure 1:**
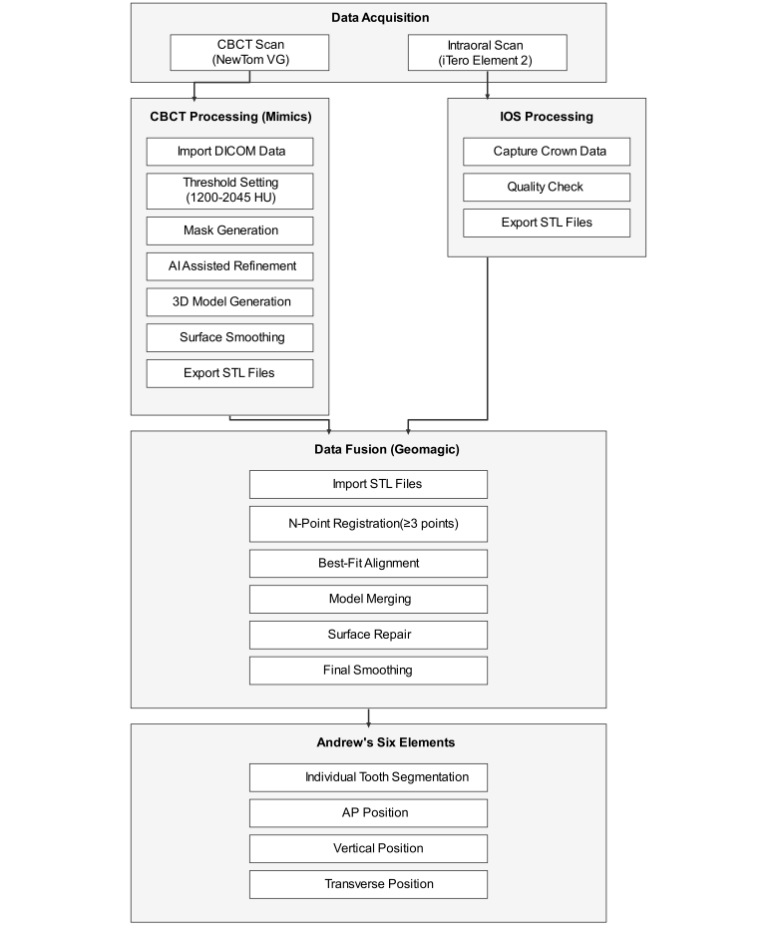
Digital dental imaging and data fusion workflow. This flowchart outlines the comprehensive workflow for digital dental imaging, integrating data from CBCT scans (NewTom VG) and intraoral scans (iTero Element 2). CBCT: cone beam computer tomography; DICOM: digital imaging and communications in medicine; STL: standard template library.

The data from the CBCT and IOS were collected and exported as digital imaging and communications in medicine (DICOM) and STL files, respectively. Within the Mimics software, the CBCT data underwent a series of processes including thresholding (1200-3045 HU), segmentation, and 3D model generation. This was followed by surface smoothing and jaw separation to create individual STL models for the upper and lower jaws. AI Edit Masks tool is used to manually correct the segmentation results, removing nondental tissues in order to reconstruct a 3D model of dental arches that contains only teeth [15]. AI Smooth tool is applied to enhance the model’s surface, while the Split tool is used to separate the jaws, allowing for individual editing and optimization of each part. The output is in STL file format. The software Geomagic Wrap is then used to fuse the data [16]. Initial alignment was achieved through N-point registration by selecting at least 3 corresponding anatomical landmarks, treating the IOS models as moving objects and the CBCT models as fixed references. Refined alignment was subsequently performed using the Best-Fit Alignment feature, with specified parameters to ensure high accuracy. The aligned models were merged using a Boolean Union operation, prioritizing the IOS data for crown detail and the CBCT data for root structures. Mesh repairs and Laplacian smoothing were applied to enhance the surface quality of the fused models, which were ultimately exported as STL files. In the final step, the orthodontic planning software was used to segment individual teeth and arrange them virtually in accordance with Andrews’ Six Elements, adjusting tooth positions and orientations to achieve optimal occlusion and aesthetics. The 3D model construction process is shown in Figure 1.

#### Patient Data Management

We ensured that all patient data were anonymized in compliance with data protection regulations and kept detailed records of all parameters and adjustments for each patient.

#### Calibration and Quality Control

We confirmed that all software versions used are compatible with each other. We were also aware that updates or changes in software versions may affect functionality. Scanners were calibrated regularly to maintain data accuracy. A quality control process to verify the accuracy of models at each step was also implemented.

#### Data Acquisition

CBCT and intraoral scans are performed to gather detailed dental data.

#### CBCT Processing

CBCT processing involves importing DICOM data, threshold setting, mask generation, AI-assisted refinement, 3D model generation, surface smoothing, and exporting STL files. IOS processing involves capturing crown data, conducting quality checks, and exporting STL files.

#### Data Fusion

Data fusion was done through Geomagic software to import STL files, perform N-point registration, best-fit alignment, model merging, surface repair, and final smoothing.

#### Analysis

For analysis, we applied Andrew’s Six Elements for individual tooth segmentation and positional analysis, including anterior-posterior, vertical, and transverse positions.

#### Interexaminer Reliability

A total of 2 independent examiners, both practicing orthodontic specialists, each with at least 5 years of clinical and research experience, assessed the samples. Their identities remain confidential in accordance with ethical guidelines. Each examiner is a certified orthodontist with a solid foundation in 3D measurement techniques and orthodontic treatment evaluation. Before data collection, both examiners underwent the same standardized training protocol to ensure uniform measurement methods, including identifying standard landmark points and using specified software tools. A consistent set of guidelines and reference standards (including validated criteria from the literature) was used, guaranteeing that the same benchmarks were applied. Each examiner scored the samples independently and under blinded conditions, with the sequence of sample numbering randomized to mitigate potential observer bias. To evaluate interexaminer scoring consistency, the intraclass correlation coefficient (ICC) was used. Bland-Altman plots were used to illustrate any differences in scores between the examiners and to evaluate the presence of systematic bias. Discrepancies in measurements were revisited and, if necessary, remeasured or discussed to improve scoring consistency.

#### Participant Grouping

Between May 2022 and May 2024, a total of 320 patients, who successfully completed clear aligner treatment at the Department of Orthodontics, Kunming Medical University Affiliated Stomatological Hospital, were invited to participate in the study. Ultimately, 136 participants met the inclusion criteria. Of these participants, 90 (66%) were female and 46 (34%) were male, 64 (47%) were adolescents, and 72 (53%) were adults. Regarding extraction status, participants were assigned to either the extraction or nonextraction group based on the clinical treatment plan established at their initial assessment. Specifically, those requiring the removal of premolars, often to address moderate to severe crowding, resolve substantial discrepancies in arch length, or correct significant malocclusion, were categorized in the extraction group (n=38, 28%), whereas those whose treatment plans did not involve premolar removal were placed in the nonextraction group (n=98, 72%).

#### Measurement and Scoring

**Figure 2:**
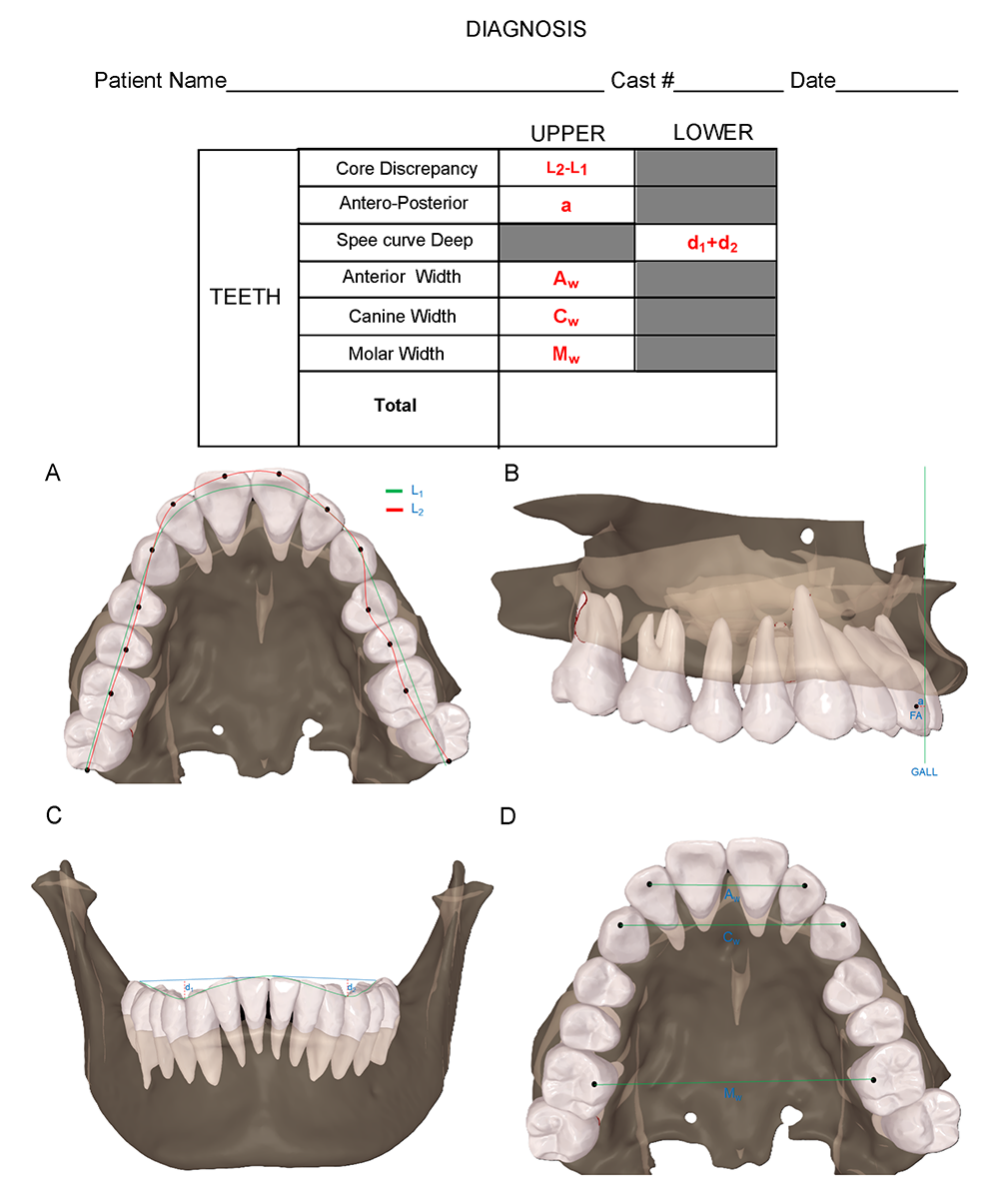
Measurement and scoring diagnostic sheet.

Based on Andrews’ Six Elements criteria [12], measurements encompass the following 6 indicators. Each indicator is scored individually, and the total score is the sum of these individual scores (Figure 2).

In Figure 2, the dental arch core line is given(A), where L_1_ is the desired length of the arch and L_2_ is the existing length of the arch. The anteroposterior position of incisors is given in B, where FA is the center point of the clinical crowns of the upper mesial incisors, GALL is an imaginary line passing through the interbrow point and perpendicular to the ground plane when in the natural cephalic position, and “a” is the perpendicular distance from the FA point to GALL. Mandibular Spee curve deep is given in C, where d_1_ and d_2_ are the depths of the Spee curves on the left and right sides, respectively. The width of the dental arch is given in D, where A_w_ is the width of the anterior teeth, C_w_ is the width of the canine, and M_w_ is the width of the molar. Upper core discrepancy (UCD) is measured from the distal of the last molar, along the central fossa of the molars and premolars, the canine cusp, and the incisor incisal edge, connecting to the opposite side. Upper incisors anteroposterior discrepancy (UAP) refers to the anteroposterior distance between the target position and the posttreatment position of the clinical crown center point (FA point) of the maxillary central incisor. Lower Spee curve deep discrepancy (LSD) is a curve formed by connecting the incisor incisal edge, canine cusp, the buccal cusps of the molars and premolars, and the distobuccal cusp of the last molar. The depth of the lowest point of the curve is measured on both the left and right sides and the 2 values are summed to obtain the Spee curve depth. Upper anterior teeth width discrepancy (UAW) is defined as the distance between the midpoints of the incisal edges of the bilateral lateral incisors. Upper canine width discrepancy (UCW) measures the distance between the midpoints of the cusps of the bilateral canines. Upper molar width discrepancy (UMW) is the distance between the midpoints of the palatal cusps of the bilateral first molars.

### Ethical Considerations

The study was registered on the Chinese Clinical Trial Registry (registration ID ChiCTR2400094304) and was granted ethics approval from the Medical Ethics Committee (approval KYKQ2022MEC0046). All participants provided written informed consent before participating in the study. No compensation of any kind was provided to the participants.

## Results

### Overview

The study received funding in May 2022, with data collection commencing in the same month, and is projected to conclude in May 2024. At the time of approval, 320 participants had been recruited, of which 136 met the inclusion criteria based on subgroup selection. The research team is now in the final phase of data analysis, having completed the advanced statistical modeling. Validation and quality checks are currently underway to ensure the accuracy and reliability of results before the study’s conclusion. Final results are to be announced by the end of 2024.

### Model Construction

**Figure 3:**
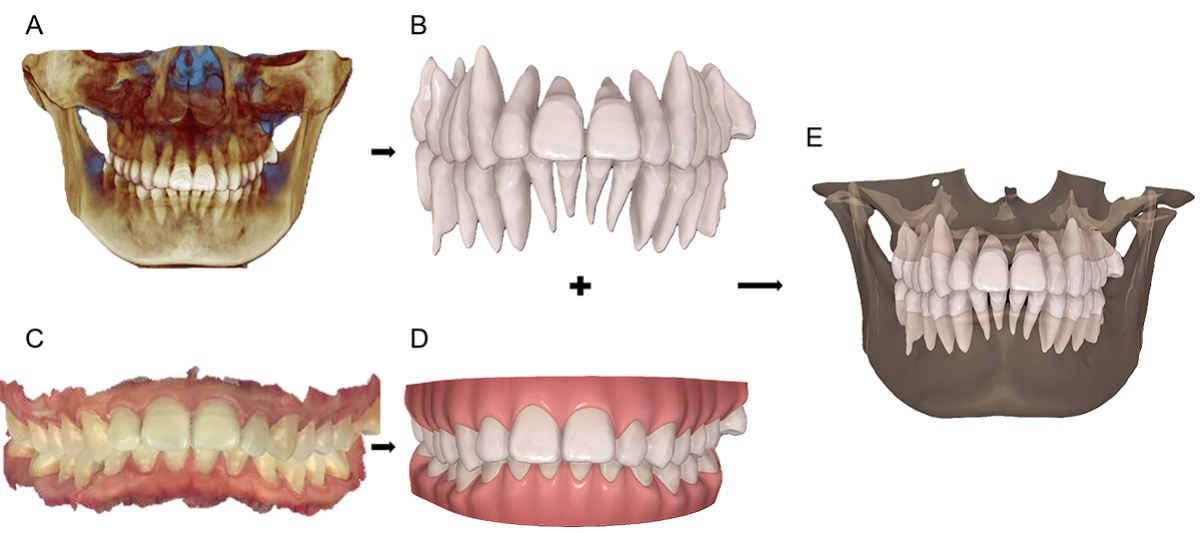
Construction of 3D fusion model.

A total of 136 patient data sets were processed to create accurate 3D dental models. CBCT and intraoral scan data were exported as DICOM and STL files, respectively. Using Mimics software, the CBCT data underwent thresholding, segmentation, and 3D model generation, followed by surface smoothing and jaw separation. The AI Edit Masks tool refined the segmentation, isolating dental tissues. Geomagic Wrap was then used for data fusion, achieving initial alignment through N-point registration and refining it with Best-Fit Alignment. The models were merged, enhancing surface quality through mesh repairs and Laplacian smoothing. Finally, orthodontic planning software segmented individual teeth and arranged them according to Andrews’ 6 Elements, resulting in a successful 3D model construction, as shown in Figure 3.

In Figure 3, the CBCT data (A) were imported into Mimics software using artificial intelligence (AI) tools to reconstruct a 3D model containing crowns and roots (B); the intraoral scan data (C) were imported into Invisalign simulator software using AI deep learning tools to reconstruct a 3D model containing crowns and gingiva (D). Then the AI tool was used in Geomagic Wrap software to combine the two to form a 3D fusion model (E).

### Interexaminer Reliability

**Figure 4:**
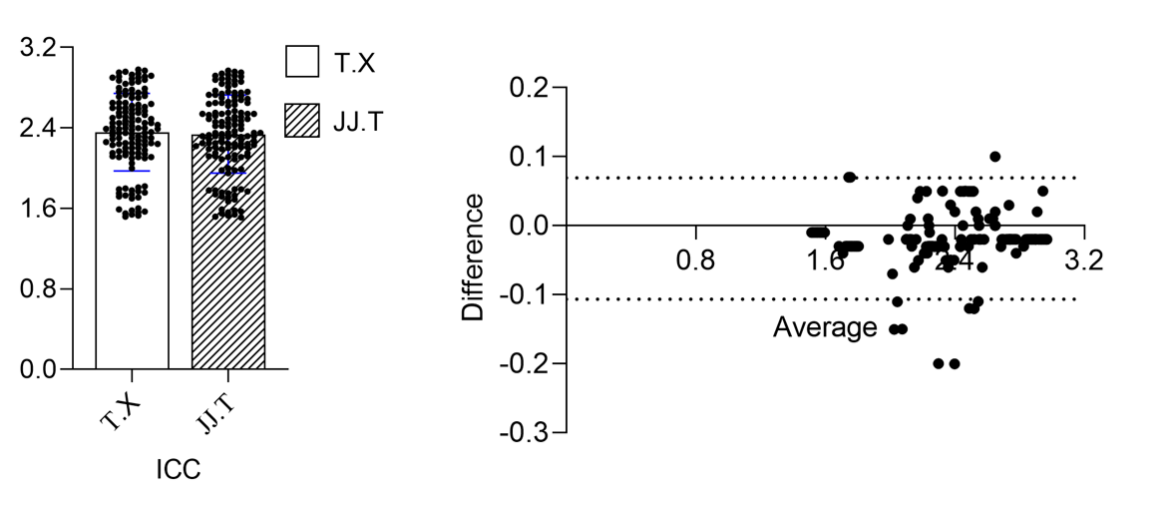
Consistency test for scores between 2 examiners.

A total of 2 independent examiners assessed the same set of measurements following the standardized protocol described in the Methods section. A total of 136 samples (from 136 participants) were evaluated, resulting in 272 total measurement sets. The overall ICC for the interexaminer agreement was 0.98 (95% CI 0.96-0.99), indicating an excellent level of consistency between the 2 examiners’ scores. A Bland-Altman plot was generated to compare the scores of Examiner A and Examiner B, visually illustrating the difference plotted against the average of the 2 measurements (Figure 4). The mean difference (bias) between the examiners’ measurements was 0.02 (SD 0.11) mm. The 95% CI of agreement extended from –0.20 mm to 0.24 mm, suggesting minimal systematic discrepancy. No trend was observed in the differences as a function of the measurement magnitude, implying that the 2 examiners were consistent across the entire range of measurements. Collectively, these results demonstrate a high level of agreement (ICC=0.98) and minimal systematic bias between the examiners across all measured parameters. The rigorous application of a uniform measurement protocol, coupled with examiner blinding and random sample order, contributed to the strong reliability indices. Consequently, the scoring system and methodology can be deemed robust for evaluating tooth movement outcomes in this study.

The data measured by 2 independent examiners are visually represented, with each dot signifying an individual measurement and the horizontal line indicating the mean. The near overlap in the distribution and mean values for both examiners suggests that their measurements are nearly identical. Furthermore, an ICC of 0.98, indicating extremely high consistency, confirms that the 2 examiners evaluated the samples in a closely aligned manner, reflecting excellent interrater reliability.

### Group and Statistical Analysis

Subjects were categorized by sex, age stage, and treatment method, with “Adolescent” defined as individuals aged 13-17 years and “Adult” as individuals aged 18-35 years (Table 1). Based on the scores, we calculated the mean and SD for individual and total scores across various groupings, using independent samples *t* tests for comparison. The confidence interval for all analyses was set at 95%. Statistical analyses were performed using GraphPad Prism (version 9; GraphPad Software, Inc).

Table 1 lists the distribution for total samples. A total of 136 samples were selected, and individuals were categorized by sex (female and male), age stage (adolescent and adult), and treatment method (extraction and nonextraction).

**Table 1:** The groups of objects (N=136).

Groups			Values, n (%)
**Sex**			
	Female	90 (66)	
	Male	46 (34)	
**Age**			
	Adolescent	64 (47)	
	Adult	72 (53)	
**Treatment method**			
	Extraction	38 (28)	
	Nonextraction	98 (72)	

### Measurement and Scoring

#### Sex Group

**Figure 5:**
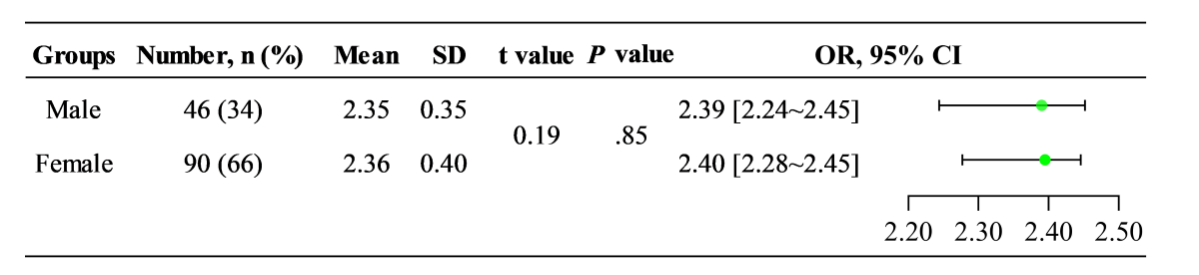
Comparison of total scores in different sex groups.

Among the different sex groups (Figure 5), the male group comprised 46 cases aged 13-35 years (mean age 21.30 years), with a total mean score of 2.35 (SD 0.35), a minimum score of 1.53, and a maximum score of 2.97. The female group included 90 cases aged 13-35 years (mean age 21.00 years), with a total mean score of 2.36 (SD 0.40), a minimum score of 1.52, and a maximum score of 2.98. A comparison of total mean scores between the 2 groups revealed a *t* value of 0.19 and a *P* value of .85, indicating no statistically significant difference.

In Figure 5, the total score distribution for sex groups is given. The male group had a total mean score of 2.35 (SD 0.35) and the female group had a total mean score of 2.36 (SD 0.40). Comparing the total mean scores between the two groups, a *P* value of .85 >.05 indicated no statistically significant difference.

#### Age Stage Group

**Figure 6:**
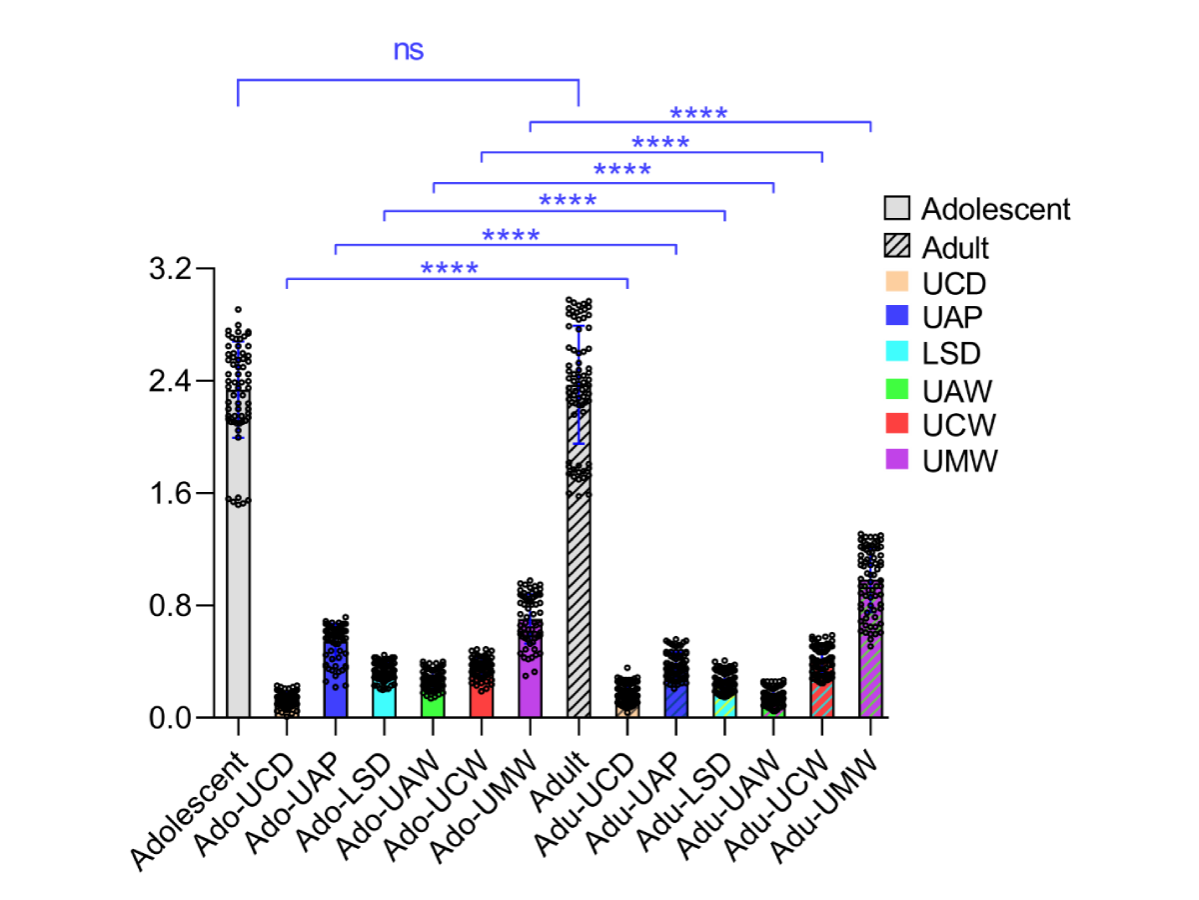
The score distribution for adolescent and adult samples. LSD: lower Spee curve deep discrepancy; UAP: upper incisors antero-posterior discrepancy; UAW: upper anterior teeth width discrepancy; UCD: upper core discrepancy; UCW: upper canine width discrepancy; UMW: upper molar width discrepancy.

**Figure 7:**
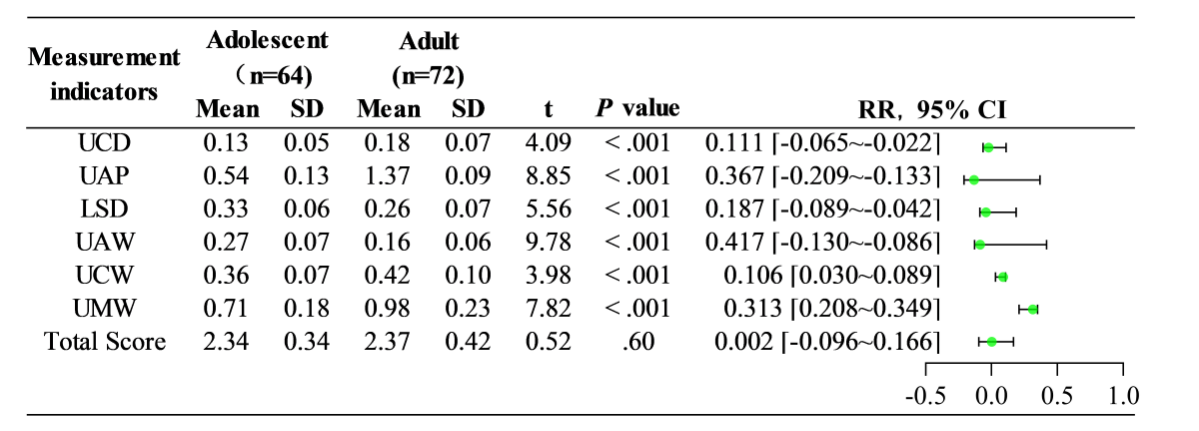
Comparison of scores in different age stage groups. LSD: lower Spee curve deep discrepancy; UAP: upper incisors antero-posterior discrepancy; UAW: upper anterior teeth width discrepancy; UCD: upper core discrepancy; UCW: upper canine width discrepancy; UMW: upper molar width discrepancy.

Among the different age stage groups (Figures 6 and 7), the adolescent group comprised 64 cases aged 13-17 (mean 13.58) years, with a total mean score of 2.34 (SD 0.34), a minimum score of 1.52, and a maximum score of 2.91. The adult group included 72 cases aged 18-35 (mean 26.53) years, with a total mean score of 2.37 (SD 0.42), a minimum score of 1.58, and a maximum score of 2.98. A comparison of total mean scores between the two groups showed a *t* value of 0.52 and a *P* value of .60, indicating no statistically significant difference. However, in the individual score items, significant statistical differences (*P*<.001) were observed in the following indicators: UCD, UAP, LSD, UAW, UCW, and UMW.

In Figure 7, the score distribution for age stage groups is given. The adolescent group had a total mean score of 2.34 (SD 0.34), and the adult group had a total mean score of 2.37 (SD 0.42). Comparing the total mean scores between the two groups (*P*>.05) indicated no statistically significant difference. In the individual score items, significant statistical differences (*P*<.001) were observed in the following indicators: UCD, UAP, LSD, UAW, UCW, and UMW.

#### Treatment Methods Group

**Figure 8:**
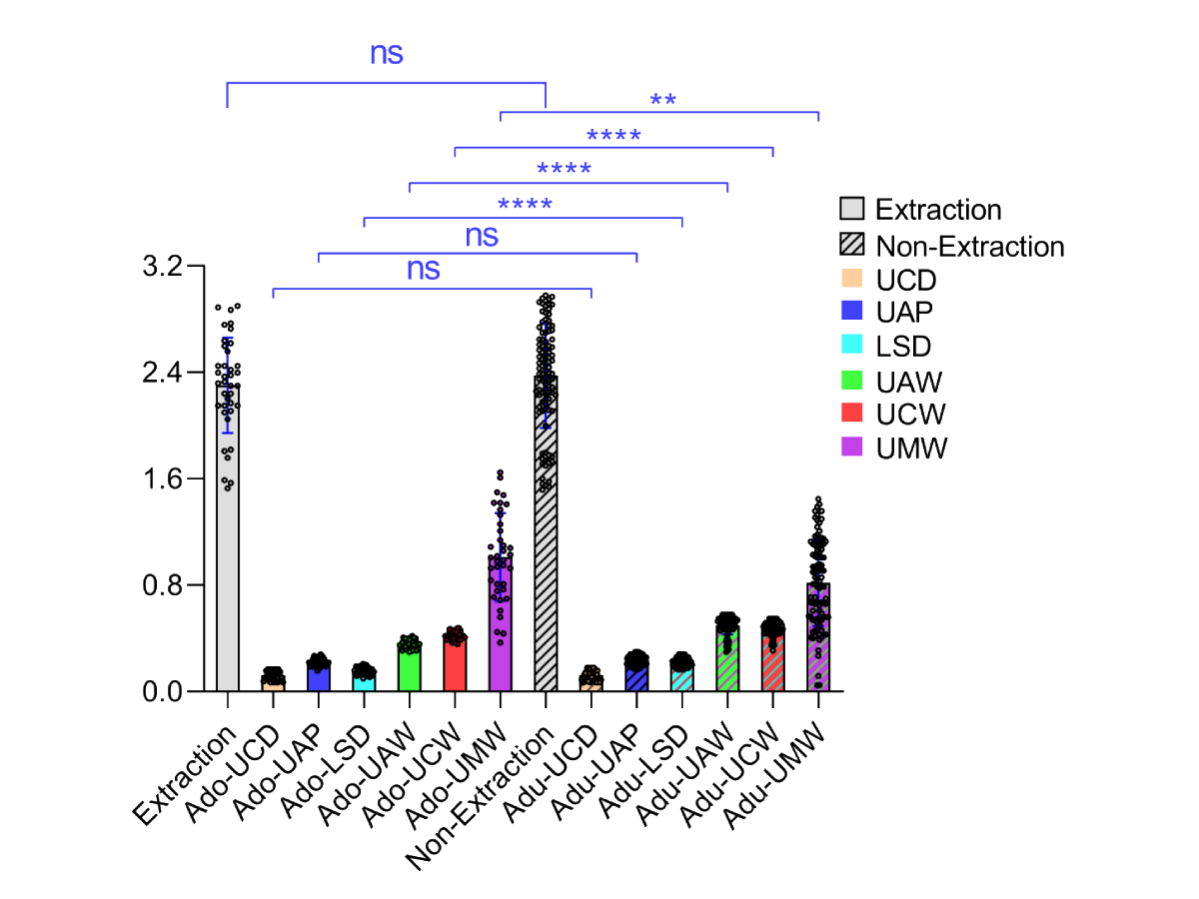
The score distribution for extraction and nonextraction samples. LSD: lower Spee curve deep discrepancy; UAP: upper incisors antero-posterior discrepancy; UAW: upper anterior teeth width discrepancy; UCD: upper core discrepancy; UCW: upper canine width discrepancy; UMW: upper molar width discrepancy.

**Figure 9:**
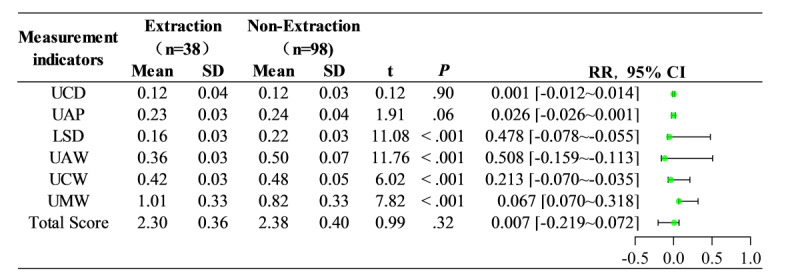
Comparison of scores in different treatment methods groups. LSD: lower Spee curve deep discrepancy; UAP: upper incisors antero-posterior discrepancy; UAW: upper anterior teeth width discrepancy; UCD: upper core discrepancy; UCW: upper canine width discrepancy; UMW: upper molar width discrepancy.

Among the different treatment method groups (Figures 8 and 9), the extraction group consisted of 38 cases, aged 13 to 35 (mean 19.74) years, with a total mean score of 2.30 (SD 0.36), a minimum score of 1.53, and a maximum score of 2.90. The nonextraction group included 98 cases, aged 13 to 35 (mean 21.63) years, with a total mean score of 2.38 (SD 0.40), a minimum score of 1.52, and a maximum score of 2.98. A comparison of the total mean scores between the 2 groups yielded a t value of 0.99 and a *P* value of .32 (*P*>.05), indicating no statistically significant difference. In the individual scoring items, there were no statistically significant differences (*P*>.05) in the comparisons of UCD and UAP. However, significant statistical differences were observed (*P*<.001) in the comparisons of LSD, UAW, UCW, and UMW.

Figure 9 demonstrates the score distribution for different treatment method groups. The extraction group had a total mean score of 2.30 (SD 0.36) and the adult group had a total mean score of 2.38 (SD 0.40). Comparing the total mean scores, UCD and UAP between the two groups (*P*>.05) indicated no statistically significant difference. In the individual score items, significant statistical differences (*P*<.001) were observed in the following indicators: LSD, UAW, UCW, and UMW.

## Discussion

### Principal Findings

Based on the findings, a 3D fusion model was successfully constructed by integrating CBCT data of the cervical and root portions with IOS data of the crown portions, using AI software in accordance with Andrews’ Six Elements Criteria. This model effectively assessed the 3D effects of clear aligners on tooth movement and provided a reliable reference for designing pretreatment target positions. The results indicated that neither sex nor age had a significant effect on total scores; however, adolescents exhibited significant differences in specific parameters, including UCD, UAP, LSD, UAW, UCW, and UMW. Similarly, while the treatment method did not significantly affect total scores, it did have a significant impact on individual items such as LSD and the buccolingual widths of certain teeth. Overall, the study validates the effectiveness of the 3D fusion model in enhancing the precision and personalization of orthodontic treatments using clear aligners, thereby establishing a new standard for evidence-based treatment planning in the field.

### Model Construction

Based on pretreatment CBCT and intraoral scans, a digital full-arch dental model integrating IOS and CBCT was constructed. 3D tooth arrangement was performed in accordance with Andrews’ Six Keys diagnostic system, successfully establishing a pretreatment 3D integrated prediction model. This model encompasses 3D images that include root and alveolar bone information. By comparing the differences between pre- and posttreatment 3D integrated models, the actual 3D tooth movement following clear aligner treatment can be examined. Baan et al [8] conducted structured light scanning, CBCT scanning, and IOS on 10 dry human skulls, achieving notable clinical accuracy in the integration of CBCT and IOS. Deferm et al [5] registered intraoral scans with CBCT for 8 dentate patients and 14 edentulous patients, finding that the average error for dentate jaws was 0.49 (SD 0.26) mm, for edentulous jaws was 0.16 (0.08) mm, and for alveolar ridges was 0.16 (0.05) mm, indicating high precision in the registration of intraoral scans with CBCT. These studies provide a theoretical foundation for the modeling used in this research, The results of this study are consistent with the above studies.

Potential limitations in model construction, particularly regarding the accuracy of the CBCT-IOS fusion technique, may significantly impact treatment planning. First, image registration errors present a major challenge. When fusing IOS data with CBCT data, discrepancies can arise, resulting in a 3D model that does not accurately reflect the patient’s actual anatomical structures [8,17-19]. Such errors may stem from slight patient movements during scanning, limitations in equipment precision, or deficiencies in the registration algorithms used. Second, differences in resolution between the 2 scanning methods can affect the accuracy of the fused model. CBCT typically provides lower-resolution images, while IOS offers high-resolution surface detail. This mismatch in resolution can lead to deviations in the detailed representation within the fused model, thereby impacting the assessment of fine anatomical structures [6,20-22]. These accuracy limitations can influence treatment planning in several ways.

#### Inaccurate Localization of Anatomical Structures

Misalignments in the model may lead to incorrect judgments regarding the positions of teeth, bones, and other critical anatomical features, adversely affecting the precision of surgical or orthodontic interventions.

#### Impact on the Formulation of Treatment Plans

Developing treatment strategies based on inaccurate models may result in suboptimal outcomes or even complications. To mitigate these impacts, it is essential in clinical practice to enhance scanning and fusion techniques. This can be achieved by using higher precision equipment, refining registration algorithms, and performing manual corrections when necessary to ensure the accuracy and reliability of the models used in treatment planning.

#### Sex

Research indicates that the sex demographic variable does not influence treatment outcomes. Xie et al [23] assessed the clinical effectiveness of SmartTrack aligners and found that sex did not affect anterior tooth rotation movement. Scott et al [24] discovered that Damon3 self-ligating brackets resulted in less discomfort during initial tooth alignment, regardless of sex. Tepedino et al [25] evaluated the predictability of the Nuvola aligner system and concluded that sex did not influence the results. Mario et al [26] investigated the accuracy of the F22 Aligner system and found that sex did not play a significant role. Taner et al [27] evaluated pretreatment dental models, lateral cephalometric measurements, and wrist x-rays, discovering that sex did not affect the outcomes of skeletal and dental cephalometric measurements. Finally, Mandall et al [6] found that sex did not influence orthodontic patients’ compliance and treatment motivation. The above research is consistent with our findings.

#### Age Stage

In this research, adolescents scored lower than adults, suggesting that adolescents may have a higher bone remodeling capacity to meet tooth movement driven by orthodontic forces. Large amounts of clinical studies suggested that adolescents experience faster tooth movement and improved treatment outcomes compared with adults, as their bone tissue responds more readily to mechanical stress, allowing for quicker bone resorption and formation. Kanou et al [28] discovered that younger individuals display accelerated rates of bone remodeling. Zheng et al [29] analyzed differences in alveolar bone support between adolescents and adults, noting that adults exhibited reduced alveolar bone support posttreatment. Kalina et al [30] assessed changes in the lower incisor alveolar bone in both adolescent and adult patients, finding that while alveolar bone thickness decreased in both groups when lower anterior teeth were proclined or retracted, the reduction was less pronounced in adolescents. Furthermore, Kuc et al [31] evaluated maxillary morphological changes resulting from incisor movement and revealed that adolescents demonstrated significantly greater maxillary remodeling capacity than adults following such movement. These results may suggest that adolescents are more effective than adults in moving teeth, a phenomenon attributed to the biological adaptability of their dental arches, which enhances the efficacy of orthodontic treatment, particularly in adjusting the transverse width of the dental arch [32]. During this phase of growth and development, adolescents exhibit higher skeletal plasticity, facilitating more efficient bone remodeling throughout orthodontic treatment.

In this research, we found that adolescents exhibit better alignment of teeth, as well as sagittal and transverse width adjustments of the incisors and molars compared with adults. This phenomenon may be attributed to the intrusion of anterior teeth along the long axis during clinical treatment, which causes the entire periodontal tissue to move coronally, resulting in a higher rate of tooth movement. Adolescents possess a stronger capacity for bone remodeling. Some researchers have longitudinally assessed changes in intercanine and intermolar widths from childhood to adulthood, discovering that both maxillary and mandibular arch intercanine and intermolar widths increase significantly between the ages of 3 and 13 years [12,33-37]. Following the complete eruption of permanent dentition, arch widths experience a slight decrease, with intercanine width decreasing more than intermolar width. The mandibular intercanine width is generally established by 8 years of age after the eruption of the 4 incisors. After the eruption of permanent dentition, clinicians should anticipate either no change or slight decreases in arch width. Although arch width changes from birth to adulthood, the magnitude and direction of these changes do not provide sufficient scientific evidence to support the expansion of dental arches beyond their established dimensions for typical patients. This is consistent with the results of this research. Nevertheless, a recent study was conducted to evaluate the effectiveness of Invisalign in reducing deep overbite in adolescent patients [38]. The findings indicated that the reduction in overlap achieved during the correction process was less than half of what the orthodontist had anticipated or planned. Furthermore, the age of the patients did not significantly influence the accuracy of overlap reduction. Notably, a trend was observed indicating a more pronounced overlap reduction in patients who did not use the bite ramp; however, this trend did not reach statistical significance.

### Treatment Methods

In this research, the extraction group scored lower than the nonextraction group, suggesting that extraction may not significantly affect effective tooth movement. Kirschneck et al [39] studied adolescents with borderline cases treated with tooth extraction and found no significant difference in the improvement of vertical relationships between extraction and nonextraction treatments in orthodontic patients. Xu et al [40] compared Chinese borderline cases with and without extraction treatment and discovered that extraction treatment may lead to differences in facial profile at the end of treatment, but no differences were observed in tooth alignment, overbite, overjet, midline, or posterior occlusion. Extraction treatment may have advantages over nonextraction treatment regarding the sagittal, vertical, and anteroposterior alignment of the anterior teeth; however, it is slightly inferior concerning the horizontal width adjustment of the posterior teeth. This may be due to extraction treatment resulting in the medial movement of molars and the distal movement of anterior teeth, which causes changes in the buccolingual width among molars, incisors, and canines. Extraction may provide additional space for adjusting the Spee curve. Elias et al [41] compared the effects of extracting premolars versus nonextraction treatment on dental arch width, contour, treatment duration, occlusal outcomes, smile aesthetics, and stability. They found that extraction led to decreased intermolar width in both arches and upper or lower lip retraction, while nonextraction treatment resulted in increased intercanine width in the lower jaw and a shorter treatment time [41]. Shafique et al [42] recruited borderline cases and found that orthodontic treatment involving tooth extraction had a significant effect on the vertical dimension. The above research results enrich this study.

### Limitations and Prospects

The limitations of this study must be acknowledged. As a retrospective study, strict inclusion and exclusion criteria were established to minimize the selection bias inherent in all retrospective investigations, but they cannot completely eliminate bias in personalized prediction models. Future research could further integrate AI technologies and use big data analysis to enhance the predictive capabilities of personalized models. While the growing demand for esthetic and modern orthodontic solutions are facilitated by technological advancements, such as 3D imaging, planning, and printing, it does not specifically address potential limitations in model construction. In particular, the accuracy limitations of the CBCT-IOS fusion technique are not discussed. These limitations can impact treatment planning by affecting the precision of digital models used for diagnosis and aligner fabrication. Inaccuracies in the fusion process may lead to suboptimal fit of aligners or misalignment in tooth movement predictions, potentially compromising treatment outcomes.

### Conclusions

The fusion model that integrates cervical and root sections from CBCT with crown data from IOS ensures highly accurate measurements and assessments of tooth positioning. This precision is crucial in orthodontics, where even minor deviations can significantly impact clinical outcomes. The use of a 3D model, facilitated by AI, enables practitioners to tailor treatments more effectively to individual patients. By validating the safe range of orthodontic movement, these models help mitigate the risk of adverse effects on periodontal hard tissues. Such stability is essential for long-term success, reducing the likelihood of relapse or complications post treatment. The study’s findings, including the adaptive differences between adolescents and adults and the effects of extraction versus nonextraction methods, provide invaluable insights. Practitioners can rely on these data-driven insights to make informed decisions that optimize individual patient care. The integration of AI and 3D modeling technology introduces new methodologies that have the potential to transform traditional orthodontic practices. This technological advancement not only enhances diagnostic capabilities but also streamlines the treatment process, making it more efficient and patient-friendly. In conclusion, the construction and application of 3D fusion models in clear aligner orthodontics represent a significant leap forward, offering substantial clinical benefits while establishing a new standard for precision, personalization, and evidence-based treatment planning in the field.

## Abbreviations

AI: artificial intelligence
CBCT: cone beam computer tomography
DICOM: Digital Imaging and Communications in Medicine
ICC: intraclass correlation coefficient
IOS: intraoral scanning
LSD: lower Spee curve deep discrepancy
STL: standard template library
UAP: upper incisors antero-posterior discrepancy
UAW: upper anterior teeth width discrepancy
UCD: upper core discrepancy
UCW: upper canine width discrepancy
UMW: upper molar width discrepancy
